# Exploring Emotions Related to the COVID-19 Pandemic through Death Education: A Qualitative Study at Italian Primary Schools

**DOI:** 10.3390/ejihpe13090139

**Published:** 2023-09-18

**Authors:** Ines Testoni, Lorenza Palazzo, Erika Iacona, Ottavia Segalla, Laura Pizzolato, Matteo Rigo, Lea Ferrari

**Affiliations:** 1 Department of Philosophy, Sociology, Pedagogy and Applied Psychology (FISPPA), University of Padova, 35139 Padova, Italy; lorenza.palazzo@unipd.it (L.P.); erika.iacona@unipd.it (E.I.); ottavia.segalla@studenti.unipd.it (O.S.); laura.pizzolato.1@studenti.unipd.it (L.P.); matteo.rigo.5@studenti.unipd.it (M.R.); lea.ferrari@unipd.it (L.F.); 2 Emili Sagol Creative Arts Therapies Research Center, University of Haifa, Haifa 3498838, Israel

**Keywords:** COVID-19, death education, children, primary school, spirituality

## Abstract

Background: The COVID-19 pandemic has impacted the lives of individuals, families, and children worldwide. In Italy, the implementation of measures such as lockdowns and distance learning in schools affected the mental health of children and families. Methods: This article employs a qualitative method to explore the efficacy of a death education project that aimed to help primary school children process the emotions and losses that they experienced during the COVID-19 pandemic. The study encompassed both the children who took part in the death education project and their teachers and parents to investigate their perspectives on the emotions of the minors and the effectiveness of the project. Results: Distance learning posed challenges for the learning process and exacerbated social inequalities. The children suffered from limited social contact with their friends and experienced negative emotions, including anger, fear, and concern for the health of their loved ones. The death education project provided a safe space for children’s emotional expression and facilitated their acquisition of coping strategies. Open communication between adults and children about illness and death proved effective in mitigating the psychological impacts of loss and preventing traumatic bereavement. Conclusion: The findings highlight the utility of death education in enhancing children’s ability to express their emotions and approach the topic of death more frankly.

## 1. Introduction

Within contemporary Western societies, there has been widespread censorship surrounding death-related matters, progressively eroding religious and traditional reflections on the afterlife. Furthermore, this censorship has made it difficult for individuals to find personal meaning in dying [1]. As posited by terror management theory (TMT), the inevitability of mortality engenders incapacitating anguish while simultaneously triggering psychological defenses that censor the determinants of this anxiety and the terror of death [2]. These dynamics can inevitably yield negative psychological outcomes in individuals’ lives [3]: The conscious removal of mortality can lead to the unconscious suppression of negative thoughts and emotions, subsequently diminishing the capacity to confront existential anxiety and thereby resulting in the impoverishment of emotions and the systematic cultural inhibition of dialogue surrounding death [4].

Additionally, while processes of attributing meaning to life and death were addressed within the family system in the past, parents now encounter difficulties providing coherent and positive responses to the issue of unrealistic death representations and are often inclined to protect children from pain and fear of death via the active avoidance of such discussions [5,6]. In light of these gaps, it is essential to plan specific educational activities that can promote reflections on existential themes and welcome concerns about death and diverse afterlife beliefs [7].

The impact of COVID-19 has led to notable effects on daily routines and mental well-being worldwide. Lockdowns and restrictions have altered sleep, eating, and tech use [8,9]. Psychological well-being has suffered, especially among vulnerable groups, such as those with prior mental issues, healthcare frontline workers, disadvantaged individuals, the elderly, and, in particular, young children [10]. Although minors may not have experienced the direct health repercussions of COVID-19, their lives have been deeply affected by social restrictions, resulting in psychological and social challenges [11]. The complete closure of schools during the health emergency, which lasted for two months in Italy and recurred in 2021, significantly impacted young children [12]. Schools had to adapt by implementing distance learning, which posed challenges for teachers who required professional development to address students’ needs [13,14,15]. In particular, minors from disadvantaged backgrounds have been disproportionately impacted by political decisions and the pandemic situation [16].

Studies have shown that over the past three years, minors experienced anxiety, depressive symptoms, sleep problems, anger, a lack of peer contact, and concerns about the present and future [12,17,18]. Social stimuli were reduced, resulting in boredom and emotional states influenced by the fears and worries of adults regarding contagion, illness, disruptions in work life, anxiety about physically distant loved ones, and the fear of loss [19,20,21,22].

Additionally, the pandemic exposed people to issues such as hospitalizations and the deaths of family members, friends, and community members. This brought the topics of death and dying to the forefront, challenging the previous societal trend of avoiding these discussions.

School-based death education programs can thus play a crucial role in supporting children by providing age-appropriate information and initiating discussions about the fear, anxiety, and suffering associated with death and dying [23]. Death education not only equips children with tools to manage anxiety but also promotes emotional attitudes, improved pain management when loss occurs, and a critical understanding of death and its universality, irreversibility, and causality [24,25]. The opportunity to discuss death and illness does not increase anxiety levels; rather, it reduces them. Engaging in these discussions allows individuals to become aware of how culture and society handle these themes and can elicit responses that are not immediately evident in everyday life [24]. Every day, families are bombarded with media information that leads children to develop a distorted and violent perception of death. Through death education and reflection on spirituality, children can address the topic of death with less anxiety [26,27]. Furthermore, following the COVID-19 pandemic and daily exposure to a sense of loss and fear of contagion, education on these topics can further support the processing of these negative experiences and children’s understanding of death [28]. Therefore, promoting death education courses can be fundamental for the mature and balanced development of children, thus preventing high-risk behaviors and potential suicidal ideation [29]. This can be achieved via the exploration of contemporary concerns about finitude, mortality, severe illness, loss, and grief [3]. This translates into participation in activities focused on sharing positive thoughts about the meaning of life [30] and traditional and innovative perspectives on the afterlife, transcendence, spirituality, and religion, thereby fostering resilience and reinforcing coping strategies to address overwhelming memories and negative feelings related to death [4,23].

The present study aimed to investigate the emotional and loss experiences associated with the COVID-19 pandemic among primary school children and to assess the effectiveness of a death education project in facilitating the processing of these experiences. Furthermore, we sought to explore the perspectives and experiences of parents and teachers regarding how the death education project facilitated the processing of emotions linked to the COVID-19 experience and the subsequent losses that they encountered.

## 2. Materials and Methods

### 2.1. Participants

The study comprised three groups: children who participated in the death education project, some of their parents, and certain teachers from the involved classes. For the group of children, snowball sampling was used, starting with a few schools with which the researchers were already in contact and based on the willingness of the school heads, teachers, and parents of the children to participate in the death education project and the research. The group of teachers and parents was selected using non-probabilistic convenience sampling based on their voluntary participation.

Eighty-four children (45 females and 39 males) between the ages of 8 and 11 (M = 9.25; SD = 0.77) in the third grade (N = 13), fourth grade (N = 58), and fifth grade (N = 13) at two primary schools in Northern Italy participated in the study.

Each coordinating teacher of the classes involved in the death education project informed the parents about the opportunity to participate freely and voluntarily in a semi-structured interview related to the project’s theme and activities. Nine parents—specifically nine women aged between 39 and 50 (M = 44.89; SD = 3.76)—chose to participate (Table 1).

Similarly, all teachers of the classes involved in the project were invited to take part, and five teachers—specifically five women aged between 42 and 58 years (M = 51; SD = 6.4)—decided to participate (Table 2). They comprised both support teachers who work with students facing difficulties and mainstream teachers who teach multiple subjects to entire classes.

This study adhered to the ethical principles of psychologists and the code of conduct of the American Psychological Association, as well as the principles outlined in the Declaration of Helsinki. A comprehensive explanation of the study objectives and analytical methodology was provided to all participants. Parents were required to provide consent for their children’s involvement in the death education project and the research. To ensure anonymity, unique identification codes were assigned to each child prior to the administration of the materials. Teachers and parents were asked their permission to record the interviews for transcription and content analysis purposes. Prior to the interviews, all participants provided signed informed consent with the assurance that the interview content would remain confidential. The Ethics Committee for Experimentation at the University of Padua granted approval for this study (reference: E9FCD1EA7D1706DB063EDCCA025DC09A).

### 2.2. Death Education Project

The children participated in a death education course that consisted of four weekly meetings of two hours each and was led by research psychologists. There were two psychologists for each class. In each session, the psychologists worked in pairs to address any potential needs of the children, such as the desire to leave the classroom momentarily if the topics being discussed became challenging for them. This possibility was explained to the children from the outset. Furthermore, the class teacher was always available in case there were specific requirements. The sessions were conducted one year after the spring lockdown of 2020, when the pandemic was still ongoing. Despite the resumption of in-person education, the children were wearing masks at school and adhering to the necessary restrictions to prevent contagion. Although the lockdown had ended some time before, the children still vividly remembered their experiences. Additionally, during the period when the death education intervention took place (March 2021), Italy was still under a state of health emergency, and many of the involved children had recently dealt with or were currently facing COVID-related experiences.

Each meeting dealt with a different topic accompanied by specific activities. Circle time, a method designed to expose emotional experiences in a circle and in a free manner mediated by facilitators, was used in the first meeting [31]: The children were invited to express their emotions and experiences in an atmosphere devoid of judgement and evaluation, allowing them the freedom to speak and share only what they desired. This tool facilitated the verbalization of their pandemic-related fears or beliefs that had not yet found a means of expression, listening, or sharing and which the children might not have been fully aware of. Following circle time, the children were introduced to other more complex (secondary) emotions and sensations. Finally, they were asked to complete a worksheet on which they could recall certain lived moments and identify the emotions they had experienced.

In the second meeting, narrations guided by stimulus images on the theme of the pandemic and distance from loved ones was used. At the conclusion of the reading, the children were given the opportunity to process their feelings, memories, or emotions elicited by the narrative, and they relied on art therapy techniques to achieve this. They were asked to express themselves via drawing, with the aim of gaining awareness of their inner world and making it visible, verbalizable, and shareable with their peers. At the end of the session, the children were provided with a guideline for an interview to conduct with their parents. Assuming the role of journalists, they were prompted to ask their parents the following questions: “We have been in a pandemic for two years now. What do you miss the most from the time before? What emotions have you experienced during these two years? What gave you the strength to move forward and overcome challenging moments during the pandemic?”

The children were encouraged to use their creativity to choose a unique way to present the answers provided by their parents in the following week (drawing, comic, story, dramatization, song, or video). The involvement of parents aimed to provide them with an indirect opportunity to engage with the topics addressed in the classroom and discuss them with their children at home.

In the next session, each child used their preferred method to share what their parents had said. Subsequently, via circle time, they were also asked the same questions to which their parents had responded. This was performed to highlight the resources that each individual used to overcome the challenging moments and negative emotions related to the pandemic. The fourth and final session was dedicated to a guided meditation activity to allow the children to visualize a safe place within themselves, as well as an inner light from which to draw strength in difficult moments. The aim of this last activity was to explore their own spirituality to make this complex concept accessible, presented as an inner strength; to be able to help in difficult moments; and to provide the energy needed to live in a positive way and stay close to people who are no longer alive. The proposed spirituality was not linked to any particular religious belief. At the end of the activity, each child was asked to draw their own inner safe place with the help of cards on which the silhouettes given by the researchers were depicted. Afterwards, sharing and final greetings were offered, during which the children were able to express their views on the project.

### 2.3. Qualitative Data Collection and Analysis

At the beginning and end of the project, the children were asked three open-ended questions before and two after the meetings to explore their opinions and emotional experiences concerning COVID-19 and to investigate the effectiveness and enjoyment of the death education project activities in which they took part. The parental interview was conducted subsequent to the death education project with the aim of exploring the children’s emotional experiences pertaining to the pandemic, the discourse surrounding the virus, and familial deaths and losses. Additionally, the aim of the parental interview was to investigate any disparities observed among the children, their level of engagement, and their perspectives on death education projects during childhood. Some examples of questions were “Has your son/daughter expressed the need to talk about the COVID-19 pandemic? Have you ever had to cope and talk to your son/daughter about the hospitalization of loved ones and/or their possible loss as a result of COVID-19?” and “What were your expectations when the project was presented? During and after the intervention, did you notice any differences at the behavioral, emotional, and/or theme level reported by your son/daughter at home?”

Conversely, the interview administered to the teachers examined the impact of the pandemic on the children, any challenges encountered within the school setting, and the emotional and social experiences of the children and their families. Moreover, it was used to gather insights from the teachers regarding the project’s influence on the children’s behavior and emotions, as well as the teachers’ opinions on the efficacy and replicability of death education projects. Some examples of questions were “During these last two years, have any particular themes emerged related to the pandemic (stress over isolation, the swabs...) or to death and loss? How were they handled?” and “What impact do you think the project had on the class? Did you notice any differences on a behavioral, emotional and/or theme level reported by the pupils in the classroom?”

The interviews with parents and teachers lasted 45–50 min each and were conducted by two of the researcher-authors of this study. To minimize the potential influence on interview content or participants’ social desirability bias, the researchers conducting the interviews were not the psychologists who led the death education activities. The interviews were semi-structured; there was a script with indicative questions and then, as each interview progressed, themes that emerged during the dialogue were explored in an unstructured manner.

A qualitative research method was employed in this study owing to its ability to accommodate the complexity of diverse perspectives and illuminate individual nuances following the Consolidated criteria for REporting Qualitative research checklist (COREQ) [32,33]. Specifically, thematic analysis, which is frequently employed in psychology studies focusing on health and well-being, was selected as the preferred method, as it enables the identification and examination of central themes within a text [34]. More specifically, an inductive approach to thematic analysis was employed, as it was deemed most suitable for identifying pertinent issues that emerged using dialogue [35]. The textual analysis of the interview transcripts, conducted by two researchers, and the children’s responses to open-ended questions were structured using six primary steps: (a) familiarization with the data; (b) the generation of codes; (c) the exploration, verification, and definition of themes; (d) the coding of data; (e) the comprehension of the emergent data; and (f) report writing. The analysis was conducted concurrently by two researchers, who compared and consolidated their findings.

## 3. Results

Eighty children out of the 84 participants in the death education project, which also comprised nine parents and five teachers, completed both the pre-intervention and post-intervention questionnaires. The qualitative analysis of the responses revealed two main thematic areas: The first concerned the emotions experienced by the children, parents, and teachers during the pandemic (Table 3), and the second related to the actual death education project (Table 4).

### 3.1. Emotions Experienced by Children, Parents, and Teachers during the Pandemic

First, children predominantly expressed negative thoughts about COVID-19 for various reasons. An illustrative summary of these thoughts included the following: “COVID makes us sick; keeps us indoors; makes us sad, bored, and ugly; and makes us cry. COVID makes us colourless.”

Other responses mentioned the use of masks; the inability to have physical contact with others; separation from relatives; confinement at home; and the highly contagious nature of COVID-19, which evoked fear. Some descriptions of the virus seemed influenced by adults or by what they had heard from the media. For instance, expressions such as “the cause of the pandemic was Mother Nature” or “it was punishment from Mother Nature because I could not see friends, go out, and live freely” were found. While most responses reflected children’s feelings of being “imprisoned”, “facing obstacles”, or being in a “trap”, some respondents expressed hope for the end of the pandemic and highlighted positive aspects of the period. These positive aspects included distance learning and having more time to sleep, reflect, and spend time with their families, allowing them to rediscover “the value of being together”. One child stated,

At first, it was bad, but then I had a lot of fun. I must say it was beautiful. No one bothered you, and you had birds singing. What could be better? You always have to find the positive side of things!

Some children described the pandemic period using terms such as “bad”, “sad”, “unpleasant”, “distressing”, and “difficult”; others mentioned feeling fear and concern; while some found it “good”, “useful”, and “interesting”. The reasons why this experience was unpleasant were tied to the characteristics of the period, such as the fact that so many people died: “Talking about it was a bit sad for me because just the thought of it broke a little piece of my heart”.

Parents also reported that their children experienced negative emotions, which were in line with those expressed by the children themselves. These emotions included fear stemming from the initial confusion surrounding the situation, which led children to enquire about the potential dangers of COVID-19 for their own lives and those of their grandparents. Additionally, children felt anger owing to the limitations imposed on their social activities. Parents also expressed their children’s deep concern for the health of their grandparents and their inability to visit relatives in the case of COVID-19 infection and hospitalization. The children also frequently asked questions such as “Mum, what if you died?”

Since children were often exposed to information from television or radio, they sought explanations from their parents. In response, the parents endeavoured to be truthful—though sometimes only partially—in their explanations. Several parents reported their happiness at having the opportunity to spend significantly more time with their families, which they would not have had during holidays.

According to the parents, the pandemic also had many negative aspects, both in terms of the children’s experiences and their psychosocial development. From the parents’ point of view, the children suffered, in particular, because of their distance from close friends and relatives. The use of protective equipment was also a source of stress and generated discussions between parents and children aimed at clarifying the reasons for their use. Lucia said the following:

As it went on, we had to remain isolated from everything and everyone, and we were not used to this. This greatly affected my daughter’s state of mind, because she felt lonely, not because she was not with us but because she could not interact in person with her classmates and the friends she usually hung out with. In addition, she suffered a lot from the distance from her maternal grandparents, where she used to spend most of the day... She missed them very much.

Physical contact, being able to play together, and attending school were what the children missed most during this period. This subsequently led some children to not develop the “instinct to approach” on social occasions, such as birthday parties, and others felt the need to create relationships.

The most stressful experiences for children were associated with internet connectivity issues, resulting in disrupted connections with their teachers and classmates, leading to frustration. Additionally, teachers often took on the responsibility of teaching, as lessons were considerably shorter during the initial lockdown phases.

Although children were not initially equipped for extensive technology use, they swiftly adapted to the new learning environment. Consequently, some parents now struggle to regulate their children’s online activities. One mother believes that owing to the pandemic and early exposure to the internet, children have entered an environment that poses potential risks for their age group.

Although most adults continued working, which undoubtedly had its advantages, it also instilled fear among parents that they might contract the virus and pass it on to their children. Consequently, parents maintained physical distance. While the rationale behind this behavior was understood, it caused discomfort for them. Giulia provided the following testimony:

My daughter would look for me, but then she realized there was a possibility I might bring the virus home, despite all precautions. It was a tough time for both of us. I tried to maintain a certain distance until the situation improved.

Several teachers reported encountering difficulties during the distance learning period, primarily because of their lack of required technological skills. Giuseppa shared her experience:

As a teacher, I faced numerous challenges. I felt thrown into a demand for technological skills that I did not possess, which forced me to invest extra hours. There were no longer any holidays; instead, every day became a working day to organize everything as best as possible, so as not to further burden these children.

Similarly, Federica stated,

I felt the need to see and interact with my students even before the start of the school day. I longed for contact because I felt disconnected from them. This desire to reconnect allowed me to embrace the computer not as a constraint but as an opportunity. Additionally, together, we made it work. Overall, it went quite well from a human and relational perspective.

For the teachers, the most challenging aspect was making relational and socialization opportunities. Whenever feasible, they provided students with avenues to express their emotions. Owing to the physical distance and associated challenges, feedback that teachers typically receive when proposing activities or new topics was limited. Even today, according to teachers, the students continue to carry knowledge gaps that the school has been unable to address. Consequently, this period was exceedingly frustrating for the teachers, despite their years of teaching experience.

While the shortcomings were more evident on the academic front, the teachers perceived that students were not “particularly stressed by this closure” on an emotional level. This could be attributed to the fact that the students’ experiences differed from the usual, including increased time spent with their parents and families. Initially, from the teachers’ perspective, the children welcomed distance learning as if it were a game. However, as they realized that this mode of learning would continue for an extended period, they began to find the situation less tolerable.

Some teachers noted that in some cases, the constant presence of parents hindered effective teaching, as parents tended to “take the place of the children”. Teachers often found themselves teaching both their students and their parents, and these children felt that they were not allowed to make mistakes as they would in a classroom setting.

The return to in-person classes was awaited with trepidation by teachers and children. Children were excited to be together and finally happy to have contact with their classmates and teachers. Federica shared her experience:

I will never forget the eyes of some of my students. When they returned in September, they looked even more beautiful than usual, with shining eyes. They communicated through their eyes, and it seemed like they were asking for a hug.

The safety measures and the thought of returning to distance learning were sources of stress and anxiety for the children. As a result, teachers had to intervene directly to reassure them, encourage reflection, and foster a sense of responsibility. Federica’s words serve as an example in this regard:

I always made an effort to showcase the small successes we had every day, also expressing, “We are in each other’s presence now [and are] no longer confined to a monitor, and that in itself is fortunate. We can see and interact in a different way, allowing for more spontaneity”. I consistently emphasized the positive aspects of the moment, resulting in them performing exceptionally well.

Considering the limited opportunities to socialize outside of school, contact with classmates and teachers played a crucial role in the students’ psychosocial development. However, there was also concern about the risk of infection during mealtimes, despite adequate spacing, as well as the fear of passing notebooks and pens, which are essential practices for teaching children to collaborate and share.

### 3.2. Death Education Project

Children’s responses to “Write a thought about COVID-19” in the pre-project and post-project periods did not reveal any differences between the two periods or the two schools. The elementary school students primarily reported thoughts with negative connotations. Conversely, mainly positive experiences could be identified from the responses to the question “How was talking to us about COVID-19 in these meetings?” In particular, the theme of children’s memories emerged: “In these meetings, I remembered many things that I experienced and heard during the pandemic”. For some children, talking about the pandemic brought to mind “bad things like not going to school and not seeing relatives, friends, and grandparents anymore”. For this reason, they felt sadness and pain, but being able to reflect on these emotions together during the EDI4APP project meetings allowed them to calm down.

The children’s responses brought out different experiences that can be grouped into main themes: a sense of relief, sharing to understand oneself and others, a sense of safety, learning new emotions, negative experiences, and positive experiences.

Many children reported that talking to the psychologists during the meetings allowed them to vent and consequently feel good, feel unburdened, and no longer think only about negative emotions related to the pandemic:

It made me feel better. I could not talk to anyone about it because I felt shy. However, with you, I could also get rid of the bad thoughts. You were the only ones I could express everything I had on my mind to. If you came back, I would probably be the happiest child in the world.

Other responses revealed the sharing dimension of the project’s activities: The children were able to talk about their own experiences and listen to others’ without feeling judged and, in some cases, found commonalities: “Talking about COVID was interesting because I discovered that my classmates were thinking similar things to me”.

One child wanted to emphasize the sense of security that the EDI4APP project focused on, stating, “It made me feel safe”. Talking about the pandemic during the four meetings allowed the children to learn and discover new things and emotions, such as empathy. It also helped them understand how to deal with the situation related to the pandemic and that “We should not give up”. In general, the feedback the children provided in response to the question “How was talking to us about COVID in these meetings?” was positive because many used the words “beautiful”, “fun”, “pleasant”, and “interesting” to describe the experience. However, some children did not like talking about this topic because it caused them to lose interest or become distressed.

The two schools that participated in the EDI4APP project were immediately supportive of the initiative. During regular lessons, the teachers frequently attempted to engage the entire class in a collective conversation to help the children share their emotions and experiences. Every Monday at the Costa di Rovigo (Ro) school, almost an hour is dedicated to this activity, and especially in the period after the lockdown, the emerging themes related to the pandemic. Roberta’s testimony is an example:

Sometimes, we talked about COVID: What happened? Have you heard the news? There are very informed children, children who do not follow any news, and children who are really informed daily about various news items—not just Italian, but about what is happening in Europe.

Even themes of death and loss had already been addressed prior to the project proposal, as some children had experienced the loss of a close relative—not because of the pandemic—or abandonment by a parent. Despite these situations, the teachers had a positive perception of the emotional state of the students. Patrizia reported,

Sometimes, we would go and ask, “How are you?” a bit cautiously. Even with Grandparents’ Day, we tried to do crafts also thinking about the grandparents who were not there, but I have to say that no child showed any particular suffering from this point of view.

Despite the concept of death and the associated fear being relative, according to the teachers, it is possible to discuss these topics with elementary school children. The key is to consider the terminology. Roberta shared her perspective: “Children naturally discuss it; some may even cry while reminiscing about deceased pets or relatives like grandparents. However, they speak about it calmly without appearing distressed. Children need to grasp the concept that both life and death exist”.

Given the teachers’ receptiveness to these issues, the expectations for the project were positive when it was introduced. The aim was to give children the opportunity to express their thoughts and emotions regarding death and loss through different means, uncovering unique insights beyond what the teachers observed on a daily basis. It also aimed to equip the school staff with tools to address these issues effectively. During the pandemic, the teachers primarily played a supportive role and refrained from asking children specific questions about their emotional experiences to avoid creating uncomfortable situations. This project revealed previously undisclosed situations, such as some children’s experiences of bereavement owing to COVID-19.

Only after explicit questioning did one child respond that they had lost their grandparent during the early stages of the pandemic. The teacher present during the encounter reported in the interview that she was surprised, as this kind of news is normally shared by the family.

Another teacher expressed her enthusiasm for participating in the project, hoping it would provide an opportunity for the children to express themselves and offer a final reflection for closure. Giuseppa shared her opinion: “At this age, children are still very self-centred. Hence, we believed this course could be beneficial in encouraging them to reflect upon their own emotions, helping them assign names and meanings to the emotions they frequently experience”.

Overall, the expectations were confirmed. However, some teachers reported that some of the words in the material given to the children were not age-appropriate, as they were too similar and not easily distinguishable. One teacher suggested paying more attention to monitoring the children’s comprehension during readings in class, using a slower narrative pace and pauses to allow for reflection.

A favourite activity for both students and teachers was guided meditation, during which the children were asked to imagine a safe place. This activity was also integrated into frontal lessons by the teachers to stimulate creativity and exposure through slides of new emotions, such as empathy and resilience, which entered some children’s vocabulary. Learning about hope, empathy, and altruism was met with enthusiasm by the children, as they realized that these affections were already part of their experience, and understanding that these were positive and fundamental dimensions was gratifying. Giuseppa reported,

When the Italian teacher reads passages that touch upon the emotions discussed during our activities, the children recognize them and say, “Ah, yes. We’ve dealt with this matter before. We already know this word, we’ve encountered it, and we’ve experienced and expressed this emotion. We’ve even created artwork based on it”.

According to some teachers, the impact of the project may not be readily observable, as none of the classes at either school exhibited significant disturbances prior to the meetings, and there were numerous variables at play. However, all interviews contained positive references to the value of these sessions, which helped the children gain awareness and opened doors for further growth. Patrizia shared her experience: “I believe there has been a heightened sense of awareness. The children were able to identify emotions that they were experiencing but could not previously label”.

One teacher mentioned the role that the EDI4APP project played in the “positive evolution from a relational and interaction point of view” that her class showed in the last period. The interviewed teachers considered the projects that address emotions, spirituality, and loss as useful for all ages, from the early years of elementary school to high schools, and they could be part of systematic education because “there are always aspects related to loss, and they are becoming more varied, more frequent, and more complex”. These paths can provide intervention and reflection tools for children, school staff, and parents. Often, teachers dealing with situations related to death and loss rely on their sensitivity and experiences, but they need external support to guide them.

The interviews revealed that children face a great deal of difficulty and shame in expressing themselves with their bodies, and a solution to this problem could be to increase the amount of school time dedicated to extracurricular activities, including art, music, film screenings, dance therapy, dramatization, or projects similar to the one proposed in this study. Roberta shared her opinion:

Unfortunately, very little emphasis is placed on these forms of expression, whereas I have seen them as crucial for many years. I see the children for who they are, and I believe we should reduce the focus on Italian or history and prioritize these types of experiences. Children require them, even if they cannot articulate it. Some children struggle with self-control and fail to regulate themselves in their interactions with others. They lack patience and often exhibit sudden outbursts.

On the parents’ side, there remains some resistance towards accepting projects that address topics such as death and loss, as not everyone is inclined to confront these issues. However, doubts can be dispelled by providing clear explanations regarding the context, objectives, and methodologies used in these interventions. Giada expressed her thoughts on this matter:

The key is to communicate effectively. This should be done prior to an activity, allowing parents a dedicated space where they can be informed about what will be done, how it will be done, and who will be involved. Familiarizing themselves with these aspects beforehand enables parents to feel more secure and engaged. Maintaining open lines of communication with families is crucial.

During the project, some of the children’s concerns emerged and were perceived by the parents. In particular, concerns related to the health of family members—that is, questions such as “Mum, but what if you die?” and “However, when will you die?”—and the impossibility of visiting relatives if they contract COVID-19 and are hospitalized. From the interviews, it emerged that many mothers agreed that their children are capable of understanding, albeit partially, the concept of death and “the pain a person can feel when losing someone they care about”. This is probably because these topics have been addressed in religious education, at school, or through experiences with animals and plants.

Many parents had positive expectations when the project was presented, hoping that it would provide a sense of closure; encourage their children to speak and highlight a reflection; help them better understand their children; help their children talk about the loss of freedom they experienced before, as well as about grief; and help their children learn to “individually face issues related to pain” and understand what happens when they experience a certain emotion. In general, these expectations were confirmed, except in cases in which the parents were unclear about the project’s objectives and in the case of a mother who expected her child to ask her questions about pain after listening to some stories or emotions shared by their classmates. Some parents initially had doubts and asked for a meeting to get clarification. The explanation that emerged from an interview about this initial apprehension was that some people probably consider death and loss to be difficult topics to address, even on a personal level.

Specifically, Martina mentioned that she initially viewed discussing issues such as isolation and detachment from society, which the children had experienced, as negative. However, after witnessing her son’s enthusiasm following the project meetings, Martina changed her perspective and recognized the project’s value in promoting sharing and camaraderie among peers. She stated,

In the beginning, I had doubts about my son’s participation, I must admit, but mainly because I did not want to reopen a burdened situation for him. When the informed consent form was presented to me, I thought, Oh no! Reinforcing this again? Despite that, I decided to allow his participation. Later on, observing his enthusiasm and how he freely expressed and shared his emotions with his classmates during the project, my opinion changed.

Although the children reported enjoying the project and expressed satisfaction at home, many parents did not observe any noticeable changes in their children’s emotional or behavioral aspects or the topics they discussed. Only Lucia noted that although her daughter had always been outgoing, she began to share her thoughts more and even express them in front of strangers after the meetings. Lucia also observed a stronger bond forming among classmates:

I have noticed that my daughter has become more open. Additionally, the fact that they started doing group work at school has been beneficial. Previously, the class was divided along gender lines, with boys sticking together and girls forming their own groups. There was no interaction between them. However, after my daughter participated in the project and they engaged in activities as a whole group, there was more bonding between boys and girls. Now, they interact, work on assignments together, and play together. In my opinion, this marks an important step forward.

From the interviews, it emerged that all parents consider education projects that address emotions, spirituality, and loss to be useful for various reasons. These topics are often not addressed within families unless prompted by specific events; therefore, giving children the opportunity to discuss them together can be particularly interesting, as they have their own language and can share their thoughts while listening to others’. One mother reported that an important factor was that the researchers conducting the meetings put themselves on the same level as the children, which made her son feel free to talk about his experiences.

These projects promote interactions that go beyond school education because they put children in touch with emotions that they experienced not only during the pandemic but also in other situations that they probably did not have the opportunity to discuss before. At the same time, it is important to recognize the limits of children in dealing with certain topics, “because children are all different, and they have different histories—even family and personal ones—and so, sometimes, someone is more sensitive”, especially if the facilitators are not capable of managing the reactions that arise. Alice expressed her opinion on this topic: “Since there’s a risk of delving so deep and touching such sensitive strings, if the activity goes so deep in the workshop, whoever is doing it must be able to manage the situation properly”.

According to the parents, it is vital for schools to systematically incorporate death education courses from early childhood and continue them over time. They believe that such courses can help prevent behavioral and relational problems that may arise as children grow, including instances of bullying.

## 4. Discussion

The study focused on two main themes: the emotional impact of the COVID-19 pandemic and the subsequent lockdown on children, parents, and teachers, as well as the implementation of a death education project aimed at primary school children. The aim of the death education project was to explore the emotional experiences and losses suffered during this challenging period. During the initial phase of the pandemic, the Italian government mandated the closure of all schools. Distance learning was proposed as a solution to continue education. However, this study revealed that distance learning posed challenges, particularly for young children, and exacerbated social inequalities related to the availability of devices and internet connection at home [36]. The use of technology for educational purposes also increased internet usage for recreational activities. Some parents struggled to regulate this uncontrolled usage and expressed concerns about children being exposed to age-inappropriate content [37]. The increased time spent online for learning exposed children to potentially harmful and violent content, along with a higher risk of cyberbullying [21]. Many parents lacked the necessary tools to support their children’s distance learning, while teachers faced difficulties owing to a lack of technological skills.

Emotional challenges were also evident in the interviews. In line with the existing literature, the lockdown period primarily affected children by separating them from their friends [18]. Moreover, the lack of physical contact and reduced school attendance led some children to prefer social distancing, while others experienced a stronger need to build relationships [37]. According to the study findings, children experienced prevailing negative emotions during the pandemic, including anger, fear, and worry, particularly regarding the health of their loved ones. Boredom, loneliness, irritability, anxiety, and restlessness were also observed during the lockdown, leading to increased externalizing behaviors [37,38]. However, amidst these negative aspects, the lockdown period presented some positives. It allowed children to sleep more, teachers to acquire new digital skills, and families to spend additional time together. Parents noted that these positive aspects improved family interactions and bonds [18]. Additionally, the collaborative relationship between schools and families improved significantly during this period, which was a novel experience for many participants [21].

The study explored the effectiveness and impact of a death education course designed specifically to help children process emotional experiences and losses during the pandemic, irrespective of whether they experienced actual bereavement. Children often worry more when they are uninformed about events around them, which can manifest in externalizing behaviors [28]. Therefore, it is crucial to acknowledge and validate children’s thoughts, feelings, and reactions while providing appropriate emotional support. Honest communication, reassurance, and explanation of the situation while considering their developmental level are essential [23].

The findings of this study indicate that a majority of the children had previous opportunities to discuss pandemic-related issues before the project meetings. Meeting with their peers was perceived as a positive and highly valued experience that allowed them to acquire new knowledge, listen to differing perspectives, express themselves freely, gain a deeper understanding of their circumstances, and find ways to cope [23]. Additionally, the theme of death and dying naturally emerged throughout the pandemic period, as children came into contact with images on television, heard stories from their family members, or experienced the illnesses or losses of loved ones owing to the virus [39].

The results highlight that both teachers and parents believe that their students and children possess some level of comprehension regarding loss and its implications. Even from an early age, children gradually develop an understanding of death, initially perceiving it as abandonment and then understanding its irreversibility before eventually recognizing their own mortality [40]. The adults who participated in this study attempted to normalize death by describing it as a natural phenomenon, different from its portrayal in video games. The existing literature suggests that communication between adults and children about illness and death has the potential to alleviate both short-term and long-term psychological effects and prevent traumatic bereavement [26,41]. Therefore, incorporating death education is crucial to enhancing children’s ability to articulate their emotions, reducing outward-oriented thinking and alexithymia, fostering attentiveness to internal dimensions, and facilitating conversations about these sensitive topics [42,43].

Moreover, according to many parents and teachers, the project demonstrated positive aspects despite not leading to specific changes in children’s behavior. This observation can be attributed, on the one hand, to the likelihood that the participating classes were already accustomed to discussing emotions (albeit not related to loss) and, on the other hand, to the limited number of meetings in a short timeframe. The interviews revealed that some parents still exhibit reluctance when addressing issues of death and loss within the family. These outcomes align with TMT, which elucidates how proximity to death elicits both a fear of death and a need to deny its significance by adopting various cultural constructs associated with immortality [44,45].

However, this study confirms the possibility of organizing interventions on these topics without compromising the well-being of participants. It was crucial to establish a welcoming environment concerning these issues. The teachers showed immediate support for implementing the project within their lessons and expressed the view that these activities are beneficial for students of all ages, from early childhood in primary school to secondary school. School staff often lack sufficient training on death and illness topics, emphasizing the importance of schools collaborating with external experts who can provide the necessary prevention tools [46,47].

One of the most popular activities that provided children with ample space for exploration was meditation for spiritual exploration. In today’s society, young people struggle to find educational paths that foster awareness of their spirituality. In the realm of death education, it is crucial to distinguish between religiosity and spirituality, enabling individuals to develop their transcendental dimension while benefiting from mindfulness, resilience, and a non-fearful approach to the topic of death, regardless of religious beliefs [44]. Numerous studies conducted during the COVID-19 pandemic have highlighted the importance of embracing the spiritual dimension as a vital tool to address illness, promote well-being, and provide compassion and empathy during periods of increased stress, distress, and anxiety [48,49,50]. Meditation has been shown to calm the mind, increase awareness, and create optimal conditions for reflection and generative thinking [51]. It enhances children’s concentration, provides them with better physiological and psychological coping mechanisms to address the pressures of modern life, and improves their mental health throughout their academic journeys [52]. Exploring one’s spiritual and transcendent dimensions from an early age not only allows individuals to form their own understanding and representation of death but also empowers children to face challenging moments, such as those caused by the pandemic [22].

## 5. Conclusions

The COVID-19 pandemic presented various unprecedented challenges for children, parents, and teachers. The implementation of the death education project discussed in this study proved to be a positive experience for both children and teachers, allowing for free expression and exploration of the spiritual dimension, particularly through meditation. This study also confirmed the possibility of providing interventions on these topics without compromising the participants’ well-being. However, the results revealed that parents often showed hesitation in addressing issues related to death and loss, although overall, they evaluated the project positively.

Therefore, it is crucial to raise awareness about the significance of discussing death and loss, which can be accomplished through the promotion of death education projects.

As a final note, we would like to point out that although there is a positive attitude within schools towards the introduction of death education courses, it is important to emphasize that most teachers have never received training on this subject. Because death education inevitably influences schools and students, it is crucial to address this gap in teacher education [47].

One limitation of this research pertains to the participants; indeed, the number of interviewed teachers and parents was low compared to the number of children reached through the death education project. Moreover, only female parents were interviewed. The inclusion of fathers would have been valuable, as their perspectives would have provided a more comprehensive understanding of family dynamics.

As the interviewees noted, the number of meetings conducted during the project was insufficient to produce observable effects on the children. Therefore, introducing similar projects in a structured manner may yield results that are more comparable to those obtained in reading education, particularly concerning emotional management and processing.

Moreover, the timeframe between the meetings and data collection was relatively short, preventing a comprehensive assessment of the project’s long-term effects. Additionally, it was not possible to conduct a longitudinal assessment to observe whether the effects of the death education project persisted over time.

Future studies could focus on creating, implementing, and analysing age-specific educational programs for different age groups of children by extending the duration and frequency of the meetings. These programs should be carefully designed to cater to children’s cognitive and emotional needs, assessing the effectiveness of such interventions in enhancing the understanding of death and managing the associated emotions.

Furthermore, a quantitative survey proposing a mixed methodology to measure changes in the variables of interest could be included.

## Figures and Tables

**Table 1:** Parents’ Demographic Characteristics.

Fictitious Names	Age	Gender	Degree	Employment
Maria	39	F	Graduate degree in nursing	Nurse
Lucia	50	F	Middle school diploma	Worker
Alice	45	F	Graduate degree in advertising techniques	Partner in a theatre company
Giulia	46	F	Graduate degree in law for business economics	Bank employee
Martina	42	F	High school diploma	Housewife
Sara	50	F	High school diploma	Employee
Anna	42	F	High school diploma	Employee
Aurora	47	F	Professional high school diploma	Employee
Paola	43	F	Accounting diploma	Employee

**Table 2:** Teachers’ Demographic Characteristics.

Fictitious Names	Age	Gender	Teaching Subjects
Patrizia	42	F	Main teacher
Federica	51	F	Main teacher
Giuseppa	58	F	Maths, movement educator
Roberta	56	F	Special needs teacher
Giada	48	F	Special needs teacher

**Table 3:** Results on the Emotions of Children, Parents, and Teachers.

Emotions	Exemplars
Children	Negative thoughts about COVID-19 (use of masks, no physical contact, separation from relatives, confinement at home, risk of contagion) o Feeling of being imprisoned, facing obstacles, or being caught in a trap o Positive aspects (distance learning; more time to sleep, reflect, and stay with the family) o No desire to talk about it (i.e., it was sad, bad, and unpleasant) o Talk about it with friends, family, and at school (i.e., it was good, useful, and interesting) o >Nightmares about COVID-19
Parents	Emotional experiences of parents o Suffering of children o Lack of physical activity o Use of technology o Fear of infection at work Emotional experiences of children o Negative emotions (fear, confusion, anger, and concern) o Positive emotions (happiness for more family time and strengthening bonds)
Teachers	During remote learning o Difficulties (technological skills, extended work hours, and maintenance of relational and socialization opportunities) o Knowledge gaps and frustration o Constant parental presence o Fruitful collaboration between school and families o Difficulties addressing children’s needs Returning to school o Positive emotions o Stress and anxiety regarding safety measures o Importance of contact with peers and teachers o Concern about the risk of contagion

**Table 4:** Results of the EDI4APP Project.

The EDI4APP Project	Exemplars
Children	o Sense of liberation o Sharing to understand themselves and others o Sense of safety o Learning new emotions o Negative and positive experiences
Parents	o Positive expectations satisfied o No noticeable changes in their children o Appreciation for death education projects o Significance of accessible death- and loss-related language for children
Teachers	o Opportunity to talk about death and loss o Positive and satisfied expectations o Enthusiasm for the project o Appreciation of guided meditation activity o Importance of involving and giving clear explanations to parents about the project

## Data Availability

The datasets used during the current study are available from the corresponding author upon reasonable request.
